# Towards a Novel Cost-Effective and Versatile Bioink for 3D-Bioprinting in Tissue Engineering

**DOI:** 10.3390/biomimetics8010027

**Published:** 2023-01-09

**Authors:** Fabian Züger, Natascha Berner, Maurizio R. Gullo

**Affiliations:** 1 Institute for Medical Engineering and Medical Informatics, University of Applied Sciences and Arts Northwestern Switzerland, Hofackerstrasse 30, CH-4312 Muttenz, Switzerland; 2 Swiss Nanoscience Institute, University of Basel, Klingelbergstrasse 82, CH-4056 Basel, Switzerland

**Keywords:** bioink, 3D-bioprinting, variable stiffness, tissue engineering, cell-environment mimicking, rheology, shear thinning, yield point

## Abstract

3D-bioprinting for tissue regeneration relies on, among other things, hydrogels with favorable rheological properties. These include shear thinning for cell-friendly extrusion, post-printing structural stability as well as physiologically relevant elastic moduli needed for optimal cell attachment, proliferation, differentiation and tissue maturation. This work introduces a cost-efficient gelatin-methylcellulose based hydrogel whose rheological properties can be independently optimized for optimal printability and tissue engineering. Hydrogel viscosities were designed to present three different temperature regimes: low viscosity for eased cell suspension and printing with minimal shear stress, form fidelity directly after printing and long term structural stability during incubation. Enzymatically crosslinked hydrogel scaffolds with stiffnesses ranging from 5 to 50 kPa were produced, enabling the hydrogel to biomimic cell environments for different types of tissues. The bioink showed high intrinsic cytocompatibility and tissues fabricated by embedding and bioprinting NIH 3T3 fibroblasts showed satisfactory viability. This novel hydrogel uses robust and inexpensive technology, which can be adjusted for implementation in tissue regeneration, e.g., in myocardial or neural tissue engineering.

## 1. Introduction

3D-bioprinting, or biological additive manufacturing (BAM), is an emerging field in biological engineering, where cells are printed directly within the hydrogel. It has the potential to mimic biological tissue closely and replicate natural tissue properties, thereby serving as a model for pharmacological assays [1,2]. The knowledge gained with pharmacological tissues may trigger future BAM advances in human tissue regeneration or replacement, and could thus meet the steady growth in demand for replacement tissue due to transplant organ scarcity. In recent years therefore both BAM instrumentation and bioink research have experienced an acceleration in development [3,4,5]. Most advances focus on increasing print resolution and guaranteeing shape fidelity and cell viability, with more versatile hydrogel formulations able to close the gap between hydrogels and natural cell environments [6]. Tuning such hydrogel properties can facilitate cell adherence and infiltration into printed structures, leading to tissue remodeling and regeneration into fully functional recovery of injured tissue [7]. Moreover the porosity of the hydrogel architecture allows improved cell-cell contact, better cell-matrix interaction and higher cell densities compared to non-porous structures [8,9], while simultaneously enhancing nutrient, oxygen and waste diffusion as well as better blood vessel ingrowth.

Researchers have used a plethora of natural and synthetic polymers and formulations in the attempt to meet the aforementioned criteria of bioinks ready for 3D-bioprinting [10]. However, most materials are not always available or affordable for tissue regeneration where larger amounts of hydrogel are necessary to print centimeter-sized tissues [11]. This can be a major drawback for certain commonly used hydrogels, such as Matrigel^TM^ or fibrin. In this work, we present a bioink based on affordable materials which are available in large quantities, and which feature all the properties needed for successful bioprinting of cell-laden scaffolds. The work is based on a combination of methylcellulose (MC) and gelatin similar to that used in food packaging [12]. Since both biopolymers show beneficial rheological properties when considered individually, a combination within one bioink could yield a 3D-bioprintable ink. Gelatin is liquid at temperatures above the yield point, ideal for cell loading and cell dispersion; while gelatin at temperatures below the yield point is ideal for shape fidelity after printing [13]. Conversely, MC is more viscous at higher temperatures, ideal for structural stability during incubation [14]. Combining these two biopolymers may thus create a bioink with a bigger printability window and one that, due to shear thinning, exerts less shear force on cells during printing. Furthermore, release of MC after incubation might result in a porous hydrogel, matching the aforementioned requirement profile of a bioink, as preliminary data (not shown here) indicates.

In addition, gentle and cell-friendly enzymatic crosslinking of gelatin after printing, which is characterized by different stiffnesses, may result in long term shape fidelity [15] and makes it available for different human tissue substitutes. This stiffness modification of gelatin, and ultimately of the bioink, is accomplished using a crosslinker, e.g., transglutaminase (TG) [16]. Thus, tunable bioink rigidity, mimicking the desired tissue type, increases the versatile application of such inks in regenerative tissue engineering.

In this work we study the combination of these two hydrogels to achieve favorable rheological characteristics for extrusion-based printing, with possible versatile implementation in regenerative tissue engineering, e.g., cardiac tissue reconstruction. Furthermore, printability of the developed bioink is characterized, elastic modulus alterations of the printed 3D constructs are examined and the ink is loaded with cells for 3D-bioprinting and long-term cell experiments.

## 2. Materials and Methods

### 2.1. Bioink Preparation

The process of bioink preparation was optimized for later bioprinting and consisted of two main components: a 15% *w*/*v* gelatin solution and an 8% *w*/*w* MC solution mixed in a 2:1 ratio. Additional 6% *w*/*v* and 10% *w*/*v* gelatin solutions were produced during the optimization process. For the gelatin solutions porcine skin gelatin (Sigma, Buchs, Switzerland) was slowly added to prewarmed $50{\;}^{{}^{\circ}}C$ DPBS (Sigma, Switzerland) and stirred until fully dissolved. This solution was stained with 0.5% *v*/*v* phenol red (Sigma, Switzerland) to help adjust the pH later. By adding 1 M NaOH (Sigma, Switzerland) as well as 5 M NaCl the pH was adjusted to physiological conditions. The final solution was sterilized in a tabletop microwave autoclave (Microjet Microwave Autoclave, Rodwell, UK), cooled to room temperature (RT) and stored at $4{\;}^{{}^{\circ}}C$ until further use. The MC solution was prepared by dissolving autoclaved MC in DPBS heated to $70{\;}^{{}^{\circ}}C$, to produce an 8% *w*/*w* MC solution. This freshly prepared MC solution was mixed while still at $70{\;}^{{}^{\circ}}C$ with the respective concentrations of gelatin solutions preheated to $70{\;}^{{}^{\circ}}C$ to blend them before the MC swells at lower temperatures. The final MC gelatin hydrogel mixture (MCG) was stirred constantly until it reached RT. Before use in further 3D-printing, the mixture was kept at $4{\;}^{{}^{\circ}}C$ for 12 h to swell.

### 2.2. Crosslinking Agent

To stabilize the printed construct mechanically a transglutaminase solution (TG) was prepared. TG solutions with varying concentrations were studied. Therefore, TG (Sigma, Switzerland) was dissolved in DPBS to yield solutions with concentrations between 60–120 mg/mL, which were sterile filtered (syringe filter Millex^®^-GS, 0.22 μm, Burlington, MA, USA) and produced freshly each time before use. To initiate crosslinking [16], the printed construct was immersed in 1 mL TG solution at RT and left for 10 min before washing with DPBS.

### 2.3. Rheology

All rheology assays were performed with an Anton Paar MCR92 (Anton Paar, Graz, Austria). The temperature-dependent complex viscosity and phase shift were assessed by oscillatory measurements at a frequency of 1 Hz. The temperature was varied in steps of $0.2{\;}^{{}^{\circ}}C$ and each step was kept for 10 s to allow for thermal relaxation. Shear thinning measurements were performed at the respective printing temperature. To ensure a homogeneous distribution of the ink between the rheometer plates, the samples were pre-sheared for 10 s at 1500 rpm. All measurements were performed in triplicate.

### 2.4. Elasticity

The elasticity of inks was measured by nanoindentation (Piuma, Optics11life, Amsterdam, The Netherlands) with probes having a spring constant of 0.025 N/m and a tip radius of 10 μm [17]. 1 mL of gelatin or MCG ink respectively was cast into 6-well plates (Greiner Bio-one, 6-wellplate, Kremsmuenster, Austria) and solidified at $4{\;}^{{}^{\circ}}C$. The enzymatic crosslinking was performed by the addition of 1 mL of the respective TG concentration, letting it rest in the incubator for 10 min. The crosslinking solution was then replaced by 1 mL DPBS and the samples were kept in the incubator until measurement. Measurements were performed in triplicate on five positions per sample, all kept at $37{\;}^{{}^{\circ}}C$.

### 2.5. NIH 3T3 Fibroblast Cell Culture

NIH 3T3 fibroblasts (ATCC, CRL 1658, Manassas, VA, USA) were cultivated in an incubator at $37{\;}^{{}^{\circ}}C$ and 5% CO${}_{2}$. The cell medium consisted of Dulbecco’s modified Eagle medium (DMEM; Sigma Switzerland) supplemented with 10% *v*/*v* fetal bovine serum (FBS, Sigma Switzerland) and 1% *v*/*v* penicillin–streptomycin (Sigma, Switzerland). The cell medium was changed every second day and cells were used after reaching confluency higher than 80%. The cells were collected by adding 1X Trypsin (Sigma, Switzerland) to the culture flask and incubated for 1 min. Cells were centrifuged for 7 min at 1000 rpm, the supernatant was discarded and cells were counted after resuspension a in fresh medium. Thereafter, the cells were ready to be used for 3D-bioprinting and cell viability assessment experiments.

### 2.6. 3D-Printing

The aforementioned bioink was printed with a RegenHU 3D-printer (RegenHU 3D Discovery, Fribourg, Switzerland) placed in a sterile environment. The designed print structure was a one or two layered 2 × 2 cm square meander with 1 mm line spacing. The design was chosen to be ready for printing into a 6-well plate (Greiner Bio-one, 6-wellplate, Kremsmuenster, Austria). The optimized print parameters were: (1) printhead/bioink temperature: $27{\;}^{{}^{\circ}}C$; (2) flow rate: 12 mm/s; (3) cartridge pressure: 25 kPa; (4) conical nozzle ID: 0.41 mm; (5) stage temperature: $24{\;}^{{}^{\circ}}C$. The bioink was prewarmed at $37{\;}^{{}^{\circ}}C$ until liquefied, 3 mL were added to the cartridge and placed in the $27{\;}^{{}^{\circ}}C$ printhead. The constructs were printed into the wells and directly covered with either DPBS, TG solution or NIH 3T3 full medium, to prevent drying out.

#### 2.6.1. Printing with Cells

3D-bioprinting was carried out following the same steps as for the ink without cells. NIH 3T3 cells were mixed into the ink before it was loaded into the cartridge to yield a 3 million cell per mL final concentration in the bioink. Enough cells were harvested in appropriate medium and centrifuged at 1000 rpm for 7 min. The supernatant was discarded, and the cells were mixed with prewarmed MCG. The cell-laden ink was immediately filled into a cartridge, which was manually turned horizontally and vertically to prevent the cells from sedimenting until the ink reached the printing temperature of $27{\;}^{{}^{\circ}}C$.

#### 2.6.2. Printability

Even though printability is a widely used term in BAM, currently there is no general agreement on when a bioink is considered ’ready for printing’ [18]. Hence, the printing evaluation relies on various parameters, for example, in extrusion bioprinting, on uniformity (U), pore factor (P${}_{r}$) and shape fidelity (I) [19,20]. In this study, the developed bioink was evaluated for these three parameters. For bioink uniformity three filaments were extruded in a single layer, at the abovementioned printing parameters and the photographs of these filaments were analyzed. Therefore, with ImageJ software (NIH, Bethesda, MD, USA), the rim of the extruded filament was measured by manually outlining it and comparing it to the theoretical length of a perfect uniform filament (Figure S1c). For calculating the pore factor, again, a double layered meander was extruded with the same printing parameters three times. With the photographs (Figure S1d) of the pores and ImageJ software, it was determined how well the printed pore area matches the theoretical pore area of a perfectly printed pore. The ratio of these values is the so-called pore factor (P${}_{r}$). Shape fidelity (I) indicates how well the printed constructs hold their height compared to the designed construct height. Thus, with the given printing parameters, a square-shaped construct was extruded three times, each with ten layers. Using the photographs (Figure S1b) and ImageJ software, the ratio of the printed height and the theoretical construct height was calculated.

#### 2.6.3. Assessment of Cell Viability

In order to verify the biocompatibility of our produced bioink, cell viability was checked at different timepoints using a live/dead assay consisting of Hoechst 33342 (ThermoFisher Scientific, Waltham, MA, USA) and propidium iodide (PI) staining (ThermoFisher Scientific, USA) [21]. Printed samples were incubated prior to fluorescence microscopy and the constructs were washed three times with DPBS. Staining was carried out for 20 min with a staining solution containing Hoechst 33342 at a concentration of 0.005 mg/mL and PI at a concentration of 0.001 mg/mL. Again, the constructs were washed three times for 5 min, each with DPBS. After that, assayed samples were imaged under a confocal fluorescence microscope (FV3000 Olympus, Tokyo, Japan). To investigate long term cell viability, the constructs were kept in the incubator and analyzed on days one, three and five after printing.

### 2.7. Image Analysis

Lenses of 4× and 10× with two fluorescence channels (Hoechst and PI) and a brightfield channel were used to capture the fluorescence microscopy images. Image stacking (channels and z-stack) was conducted by Fluoview software (FV21S-SW, Olympus, Japan). Using ImageJ software (NIH, Bethesda, USA), cell viability was further evaluated by converting the images to 8 bit gray value format, adjusting brightness and contrast values and threshold values [22]. Cell viability of each fluorescence image was calculated with the following formula:

(1) $$\mathrm{cell}\;\mathrm{viability}\;\mathrm{in}\;\%=\frac{100\times \mathrm{number}\;\mathrm{of}\;\mathrm{alive}\;\mathrm{cells}}{\mathrm{number}\;\mathrm{of}\;\mathrm{all}\;\mathrm{cells}}$$

Next, the average cell viability of each timepoint was calculated by analyzing five randomly selected fluorescence images at each timepoint.

## 3. Results

### 3.1. Rheological Assessment of the Bioink

**Figure 1:**
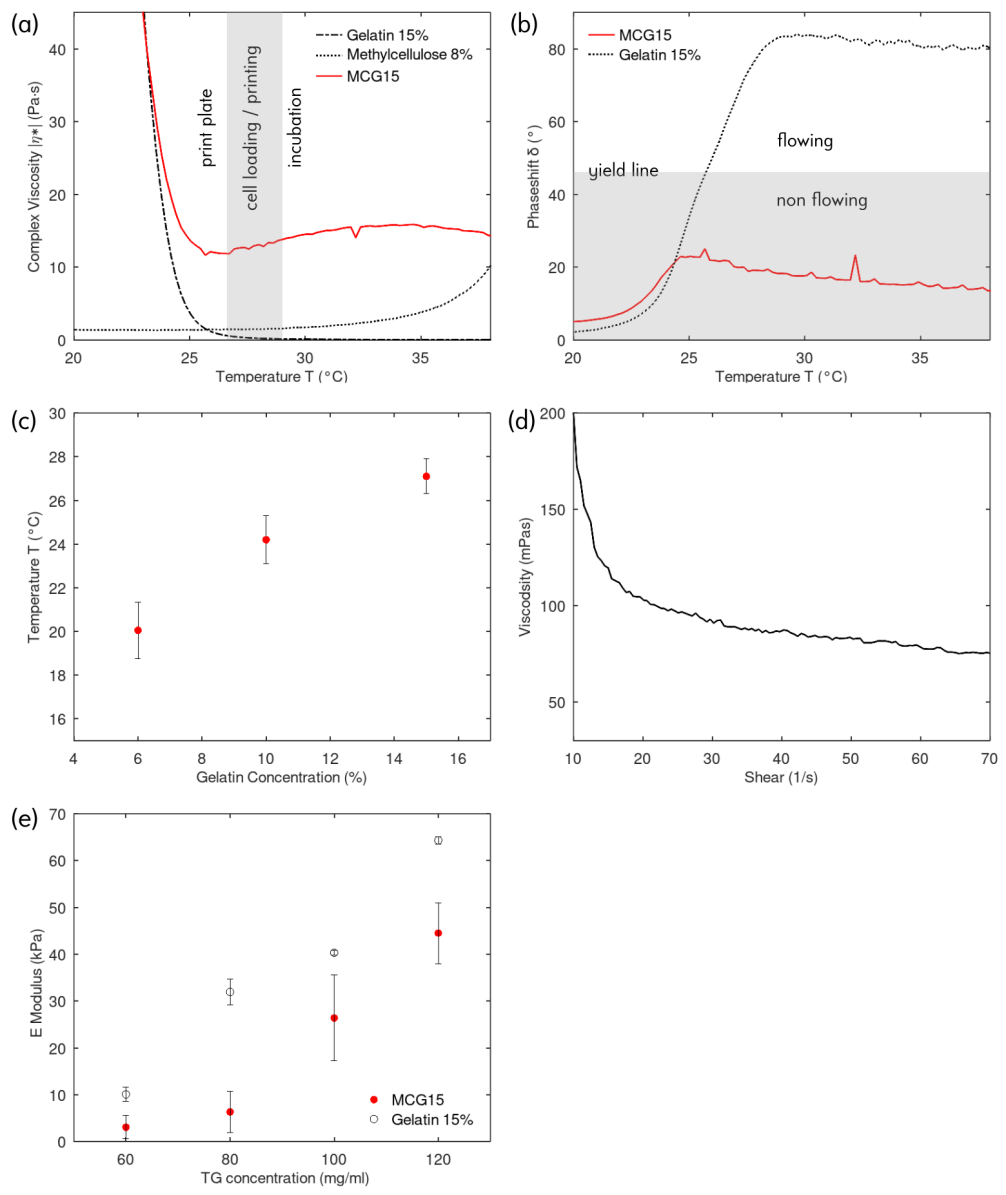
(**a**) Complex viscosity of 15% gelatin, 8% MC and MCG15 in function of temperature, showing the three working regions: (1) instant gelation on the print plate; (2) low viscosity for cell loading and printing; (3) slightly higher viscosity for structural stability at incubation temperatures; (**b**) temperature phase shift during oscillation rheology measurement showing flow and non flow regions for 15% gelatin and corresponding MCG15 ink; (**c**) Yield point in function of gelatin concentration in MCG, e.g., MCG6, MCG10 and MCG15; (**d**) Shear thinning curve of MCG15; (**e**) Nanoindentation elasticity measurements for 15% gelatin and corresponding MCG15 ink for various TG crosslinking concentrations.

The rheological properties of bioinks are crucial for successful extrusion as well as for achieving structural stability of the printed constructs. To reduce shear stress exerted on cells during extrusion, bioinks will typically show shear thinning and liquify during the printing process. Biomaterials with temperature-dependent viscosity can be used to render the ink liquid and further reduce the stress on cells. Gelatin is often used in that context, although it has a sharp viscosity transition between solid and liquid (see Figure 1a,b). The low viscosity at liquid state renders the gelatin difficult to print in a controlled manner and additional biomaterials are therefore necessary to increase the viscosity. In our approach, we blended MC with the gelatin base. As shown in Figure 1a, MC has a low viscosity at temperatures below $30{\;}^{{}^{\circ}}C$ with a slight increase towards higher temperatures. Mixing MC and gelatin will form a material with two phases. As gelatin is intended to be the cell carrier material which will be enzymatically crosslinked, it is necessary that the gelatin phase is connected to ensure structural stability. Therefore, a ratio of 1:2 (MC:gelatin) *v*/*v* was chosen.

The rheological properties of the methylcellulose-gelatin (MCG) ink are shown in Figure 1a. Three regions of interest are discernable: (i) print plate—the temperature region driven by the gelatin phase, the complex viscosity increases strongly towards lower temperatures. The printing plate will be set at this temperature to solidify the ink immediately upon extrusion; (ii) cell loading and printing—the temperature range where gelatin and MC have the lowest complex viscosity. This range is suitable for extrusion at low shear forces and for suspending the cells in the ink; (iii) incubation—in this temperature region, the MC phase slightly increases the complex viscosity (Figure 1a) and further reduces the ability of the MCG ink to flow (Figure 1b). This property is exploited to maintain a stable structure during the enzymatic crosslinking in the cell incubator at $37{\;}^{{}^{\circ}}C$.

Figure 1b shows the temperature-dependent MCG and gelatin phase shift for the oscillation rheology measurement. Hydrogels with a phase shift above $45^{{}^{\circ}}$ are considered flowing, whereas inks with a phase shift below $45^{{}^{\circ}}$ are defined as non flowing and thus able to retain their shape after extrusion printing. Gelatin shows a clear transition between the two regimes and the presence of a yield point (temperature at $45^{{}^{\circ}}$ phase shift). In comparison, the MCG ink remains non flowing over the measured temperature range. In Figure 1b, the concentration of the gelatin ink and the gelatin phase contained in the MCG ink are the same, i.e., 15%. Hence, it can be seen that the MCG ink follows the behavior of the gelatin at temperatures below the yield point, whereas above, the rheological influence of MC becomes dominant. When cooling below the yield point, the phase shift quickly drops towards zero indicating fast transition from non flowing viscous to a solid gel behavior. This yield point is mainly influenced by the gelatin concentration as shown in Figure 1c. The MCG solid gelation point can be tuned depending on the application. At the yield point, the complex viscosity is lowest and therefore, printing and cell-loading is best performed at this temperature. To reduce the thermal stress on cells during printing, the MCG15 ink which has the highest yield point temperature ($27{\;}^{{}^{\circ}}C$) was chosen for the following experiments. The shear thinning measurement of MCG15 at $28{\;}^{{}^{\circ}}C$ (Figure 1d) indicates that the MCG15 ink retains the shear thinning properties of MC.

### 3.2. Mechanical Characterization

Mechanical properties of the bioink are important for cell function and cell fate since human tissues are characterized by different stiffnesses [23]. Hence, tuning the degree of stiffness of our bioink was performed by submerging cast gelatin 15% or MCG15 in 1 mL of different concentration TG solutions for 10 min. TG can catalyze the crosslinking bond formation in gelatin [16]. Subsequently, Young’s Modulus was determined by nanoindentation. As shown in Figure 1e), Young’s Modulus of gelatin hydrogels increased with increasing TG concentration in the crosslinking solution and the same is true for our produced MCG15 bioink: the higher the TG concentration, the higher the Youngs’s Modulus of the crosslinked cast. Compared to the corresponding pure gelatin casts, the values are lower, but the ability to tune still seems to be possible. The lower values can be explained by the overall lower gelatin concentration in the mixture and, hence, fewer crosslinking events. Furthermore, it could indicate a more porous structure, due to the uncrosslinked MC. For the cell experiments, a concentration of 120 mg/mL TG was chosen to obtain an E-modulus suitable for fibroblast cell culturing.

**Figure 2:**
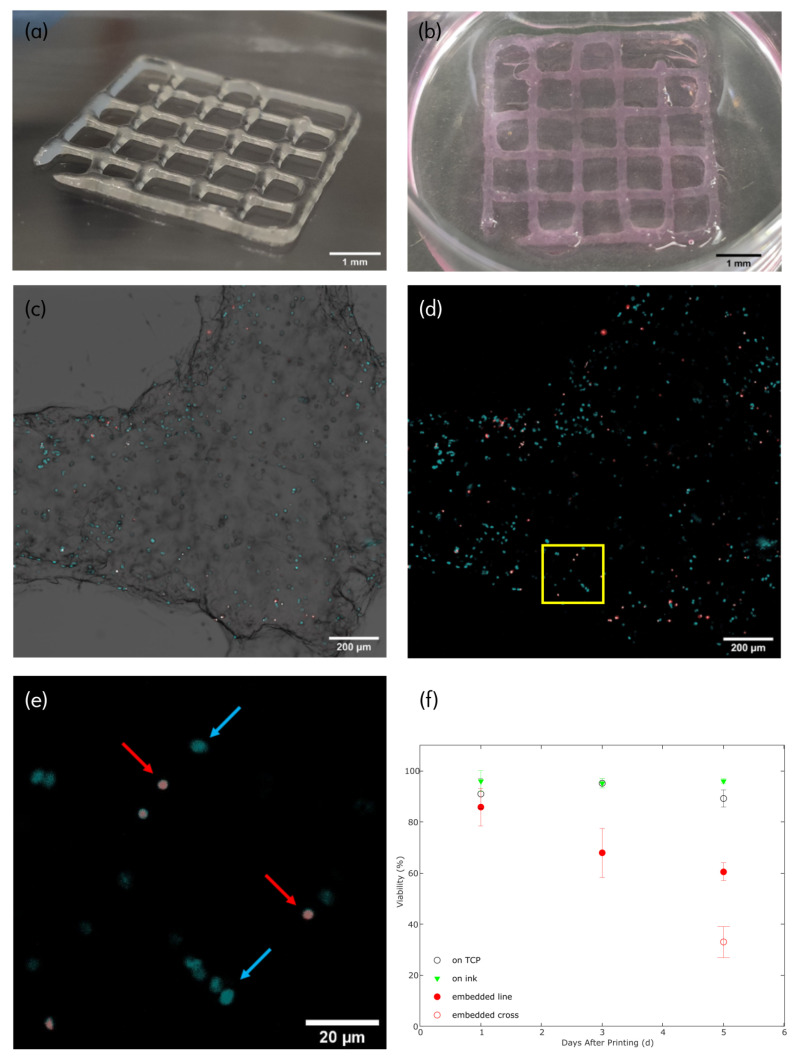
(**a**) Photograph of a 3D-bioprinted construct directly after printing and submersion for 5 min in a corresponding TG crosslinking solution. Ink contains NIH 3T3 cells. A photograph of a non crosslinked construct directly after printing can be found in the Supplementary Data Figure S1a, scale bar = 1 mm; (**b**) Photograph of a 3D-bioprinted construct five days after printing and incubation at 37 °C, ink contains NIH 3T3 cells, scale bar = 1 mm; (**c**) Fluorescence microscopy image of 1 d postprint construct stained with Hoechst 33342 (blue) and PI (red), stacking of BF, Hoechst and PI channel, scale bar = 200 μm; (**d**) Fluorescence microscopy image of 1 d post-print construct, stacking of Hoechst and PI channel only, scale bar = 200 μm; (**e**) Zoom into fluorescence microscopy image of 1 d post-print construct. Blue arrows indicate Hoechst 33342 staining of cell nucleus (live cell), while red arrows indicate PI staining of cell nucleus (necrotic cell), scale bar = 20 μm (**f**) Graphic cell viability illustration of cells seeded on tissue culture plate (TCP, control I, empty black circle), of cells seeded onto previously 3D-printed constructs (control II, green triangle) and cells 3D-bioprinted within the bioink (red circles). Additionally, on day 5, the viability is split into viability in crossing structures (empty red circle) and viability in ’line’ structures (full red circle), all with corresponding error bars.

Besides mechanical stability of gelatin and its mixture by TG crosslinking, thermal stability is also reported to be influenced. According to previous work by Liu et al. [24], crosslinking increased thermal stability in the MCG ink as well. Neither non-crosslinked pure gelatin nor non-crosslinked MCG ink held its form after incubation, while the crosslinked counterparts were stable over at least 5d of incubation, making them applicable in long term cell culture experiments (Figure 2b).

### 3.3. 3D-Bioprinting

Based on the rheological data, 3D-bioprinting with MCG15 was carried out. The best printing results were achieved at $27{\;}^{{}^{\circ}}C$ bioink temperature, $24{\;}^{{}^{\circ}}C$ ambient temperature, 12 mm/s feeding rate, 25 kPa printhead pressure and a conical nozzle with an ID of 0.41 mm. With these parameters, reproducible and accurate printing of cell-loaded constructs was possible (Figure 2a and Figure S1a). Extruded filaments had a width of 0.8 mm and a height of 0.25 mm. Cell dispersion within the MCG15 ink was achieved through resuspension of the centrifuged cell pellet and manual horizontal and vertical movement of the bioink-filled cartridge while cooling to print temperature. Loading the ink with cells did not alter the printability of the bioink and fluorescence microscopy images (Figure 2c,d) confirmed the even distribution of cells within the 3D-bioprinted construct. A movie of the corresponding printing process can be found in the Supplementary Data (Movie S1).

#### Printability

Printability of the developed bioink was analyzed by determining uniformity (U), pore factor (P${}_{r}$), and shape fidelity (I), respectively. All measurements were made on a one-, or ten-layered construct with the same printing parameters, listed in Section 3.3. Uniformity measurements of the developed bioink displayed a very high uniformity of a one-layered filament: U = 1.012 ∓ 0.004, (n = 6). Pore factor (P${}_{r}$) of a one-layered construct was calculated to be P${}_{r}$ = 0.86 ∓ 0.10, n = 7. A ten-layered square construct was printed and used for determining the shape fidelity (I) of the print compared to a corresponding designed control construct. It was determined to be I = 0.93 ∓ 0.05, n = 6.

### 3.4. Cell Viability

Cell viability within the 3D-bioprinted construct was evaluated after one, three and five days post-printing. At each timepoint, the cells were stained and a dead/alive image analysis was conducted (Figure 2e). As shown in Figure 2f, high viability was measured for the NIH 3T3 cells at early time points. One day after printing, the average cell viability within the construct was 86%. After three days post-printing, cell viability dropped to 68%. At day five, a difference was observed in the viability of cells located in regions where the print lines cross (crossing structures) and for cells elsewhere (’line’ structures). The viability for cells on the crossing points dropped to 33%, whereas in cells elsewhere, only a slight drop to 61% was observed, indicating a deficiency in nutrient diffusion in the thicker cross point regions. Additional experimental data, studying the viability in single-layered constructs, showed that after 5 d in culture, the cell viability is still at 82% (Supplementary Data Figure S2), supporting the hypothesis that lower cell viability in a thicker construct is due to nutrient shortage.

Control measurements of cells seeded into the same culture well as a previously 3D-printed construct (without cells in the MCG ink) yielded 96% (day 1), 98% (day 3) and 96% (day 5) viability, indicating the high cyto-compatibility of the MCG ink. Meanwhile, another control of solely NIH 3T3 cells seeded on tissue culture plastic (TCP) yielded a 91% (day 1), 95% (day 3) and 89% (day 5) viability.

## 4. Discussion

The hydrogel preparation method applied produced MCG bioinks, which showed stable and repeatable rheological behavior immediately after preparation. Repeated shear stress assessments at constant temperatures produced replicable results, independent of the number of repetitions. In contrast, repeated temperature ramps induced a change in rheological behavior, indicating that the phase-separation of gelatin and MC varies with temperature cycles. Such temperature variations may be intentionally applied or can arise when moving the ink from $4{\;}^{{}^{\circ}}C$ storage to the warm print head several times. It is therefore recommended always to prepare the inks freshly before use and to keep track of the times the ink changes temperature.

Preliminary results (not shown here) indicate that the preparation protocol strongly influences the spatial phase distribution of gelatin and MC, which ultimately also influences the porosity of the crosslinked gelatin scaffold once the MC is removed. In our work we chose a 2:1 ratio of gelatin: MC in order to achieve an interconnected and structurally stable gelatin network after solidification. To optimize porosity, more investigations of mixing ratio as well as other parameters have to be performed. This is of importance for the precise engineering of the diffusion distance and transfer rate of nutrients to the embedded cells as well as the macro stiffness of the scaffold. Further investigation of these aspects are beyond the scope of this paper, however.

Scaffold stiffness is a central parameter in tissue engineering since it influences the differentiation and maturation of cells, e.g., cardiomyocytes differentiate and mature best when embedded in a matrix with E-modulus of around 11 kPa [25], while neuronal cells prefer scaffolds with 20 kPa [26]. The stiffness of the MCG bioink can be tuned by the concentration of TG. To a certain extent, this can be accomplished independently of the gelatin concentration, which in turn can be varied to optimize the printing parameters, i.e., gelation temperature, printhead and substrate temperature. The independent control of these two characteristics enabled by enzymatic crosslinking opens the door to a wide range of applications. Compared to harsh UV crosslinking, enzymatic crosslinking is less harmful to cells and its rate depends on the reagent concentration. However, enzyme activity can be reduced by components present in the culture media or scaffolds, e.g., serum and growth factors. This limits the application of the MCG bioink.

With the rheological data at hand, optimal printing parameters could be determined: $27{\;}^{{}^{\circ}}C$ was ideal for both printhead and bioink temperature. At this temperature, the MCG ink had a good ability to flow and the overall printing pressure on the cells could therefore be minimized. The cooler printing substrate ($24{\;}^{{}^{\circ}}C$) led to fast solidification and, hence, good print accuracy. This accuracy was supported by the characterization of the bioink’s printability. The measured factors (uniformity, pore and shape fidelity) were used to quantitatively determine how close experimental (printed) filaments, pores and construct heights match the theoretical design. Uniformity showed good values (∼1) while pore factor and shape fidelity were a bit lower, indicating an overly gelled bioink immediately after printing.

Bigger filament widths and heights compared to the used nozzle can be explained with a certain filament collapse during printing. Further investigations into better filament stabilization needs to be carried out to achieve higher print accuracy. To a certain extent, more precise structures could have been printed (lower line spacing) with the same printing parameters, but, for the existing cell experiments, no higher precision was necessary.

As shown in Figure 2f, the printing process has a minor influence on cell viability since one day post-print viability is 86%. This leads to the conclusion that our optimized printing parameters, e.g., print temperature, flow rate, print speed and print pressure, are sufficient for printing cells with high viability, high accuracy and low shear stress exerted on the cells during printing.

Long-term cell viability analysis showed a high value for early timepoint (1 d) of around 86%, whereas this value dropped after three days to 68% and five days post-printing to 61% or 33%. We suggest that the major impact on viability is the lack of proper nutritional exchange since, as shown in Figure 2f, cell viability in a crossing structure (thicker structure) drops dramatically (33%) compared to that in a ’line’ structure (61%). This can be explained by the fact that cells have to be in close vicinity to a blood capillary to be optimally provided with nutrients and gas diffusion [27]. Cells within a crossing structure are further away from nutritional exchange between them and the medium, leading to higher cell death. Additional experimental data of single-layered 3D-bioprinted constructs, with high viability (>80%) over 5 days in culture, supports this assumption. Further investigation into the reason for decreased cell viability after several days of incubation and the nutrient proximity issue needs to be carried out. Nevertheless, controls of cells seeded onto an already-printed construct, without cells in the bioink, had a very high viability of between 96–98% for all days of culture. Therefore, our bioink shows very good intrinsic cytocompatibility.

## 5. Conclusions

The present study demonstrates the ability to 3D-(bio)print tissue mimicking constructs with widely available and cost-effective components, such as gelatin and MC. The MCG15 bioink displays favorable printing behavior, as demonstrated in the rheological assessment. The gelatin & MC combination widens the printability window by increasing the yield point to more physiological temperatures, while solidifying fast at slightly lower temperatures. Therefore, shear stress as well as temperature stress on the printed cells can be limited, shown by one day post-print cell viability assessment. Printability characterization showed uniform strands, satisfactory structural integrity and square pores. Furthermore, since scaffold stiffness is of paramount importance in cell differentiation and maturation [28,29], it was shown that elastic modulus adjustments of the novel bioink were possible by crosslinking the printed construct with TG, enabling it to biomimic cell environments for different tissue types. Paired with the high intrinsic cytocompatibility, the novel ink has the potential to be used in future tissue engineering applications, e.g., regenerative medicine, in-vitro testing or disease modeling.

## Data Availability

No data other than presented in the manuscript or Supplementary Material were created or analyzed in the presented work. Data sharing is not applicable to this article.

## Supplementary Materials

The following supporting information can be downloaded at: https://www.mdpi.com/article/10.3390/biomimetics8010027/s1, Figure S1. (a) Photograph of a 3D-printed construct directly after printing without submersion in a TG crosslinking solution. Ink contains no NIH 3T3 cells, scale bar = 1 mm; (b) Photograph of a ten-layered 3D-printed construct directly after printing, without crosslinking for shape fidelity measurements. Ink contains no NIH 3T3 cells, scale bar = 1 mm; (c) Micrograph of a construct directly after printing, without crosslinking for uniformity measurement. The red line depicts the rim of the extruded filament, and its length is compared to a theoretical perfectly uniform filament. Ink contains no NIH 3T3 cells, scale bar = 1 mm; (d) Micrograph of a pore within the construct directly after printing, without crosslinking for pore factor (P_*r*_) measurement. Ink contains no NIH 3T3 cells, scale bar = 1 mm; Figure S2. (a) Graphic cell viability illustration of cells 3D-bioprinted within the bioink (red circles) in a one-layered construct over a period of 5 d in cell culture. All viabilities are above the 70% threshold of the ISO 10993-5-2009 for biocompatibility. All with corresponding error bars; Movie S1. A video showing a short sequence of the printing process.
