# Supplementary material for: Towards a Novel Cost-Effective and Versatile Bioink for 3D-Bioprinting in Tissue Engineering
Source: Biomimetics (Basel). 2023 Jan 9;8(1):27. doi: 10.3390/biomimetics8010027 (PMC9844313; doi:10.3390/biomimetics8010027)
Supplement: Supplementary file 1 [file biomimetics-08-00027-s001.zip › biomimetics-2068446-supplementary-final.pdf]

Supplementary Data

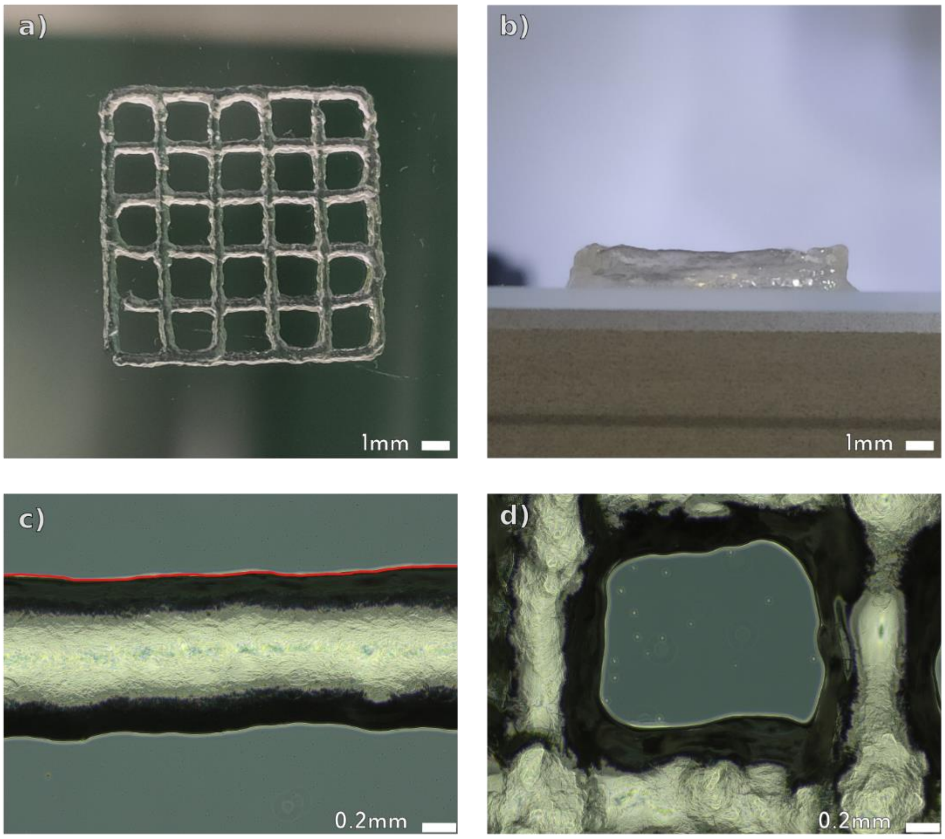

Figure S1. (a) Photograph of a 3D-printed construct directly after printing without submersion in a TG crosslinking solution. Ink contains no NIH 3T3 cells, scale bar = 1 mm; (b) Photograph of a ten-layered 3D-printed construct directly after printing, without crosslinking for shape fidelity measurements. Ink contains no NIH 3T3 cells, scale bar = 1 mm; (c) Micrograph of a construct directly after printing, without crosslinking for uniformity measurement. The red line depicts the rim of the extruded filament, and its length is compared to a theoretical perfectly uniform filament. Ink contains no NIH 3T3 cells, scale bar = 1 mm; (d) Micrograph of a pore within the construct directly after printing, without crosslinking for pore factor (P_r) measurement. Ink contains no NIH 3T3 cells, scale bar = 1 mm.

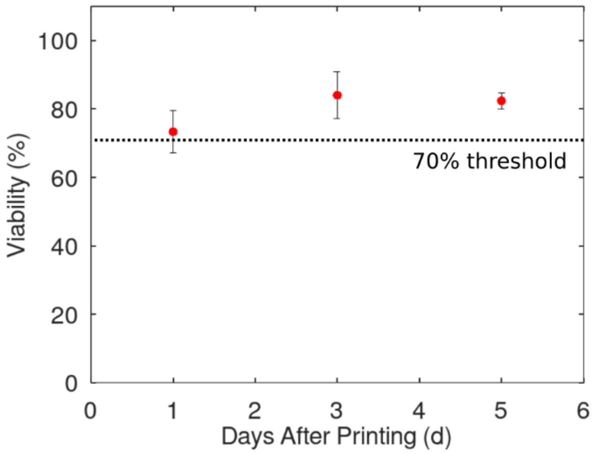

Figure S2. (a) Graphic cell viability illustration of cells 3D-bioprinted within the bioink (red circles) in a one-layered construct over a period of 5d in cell culture. All viabilities are above the 70 % threshold of the ISO 10993-5-2009 for biocompatibility. All with corresponding error bars.

Movie S1. A video showing a short sequence of the printing process.
